# HPV Infection in a Cohort of HIV-Positive Men and Women: Prevalence of Oncogenic Genotypes and Predictors of Mucosal Damage at Genital and Oral Sites

**DOI:** 10.1155/2013/915169

**Published:** 2013-03-05

**Authors:** Giulia Marchetti, Laura Comi, Teresa Bini, Marco Rovati, Francesca Bai, Barbara Cassani, Marina Ravizza, Marco Tarozzi, Alessandro Pandolfo, Serena Dalzero, Enrico Opocher, Solange Romagnoli, Antonio Carrassi, Silvano Bosari, Antonella d'Arminio Monforte

**Affiliations:** ^1^Department of Health Sciences, Institute of Infectious and Tropical Diseases, San Paolo Hospital, University of Milan, Via A Di Rudinì 8, 20142 Milan, Italy; ^2^Surgery Chair, Department of Health Sciences, San Paolo Hospital, University of Milan, 20142 Milan, Italy; ^3^Pathology Unit, Department of Health Sciences, San Paolo Hospital, University of Milan, 20142 Milan, Italy; ^4^Department of Health Sciences, Institute of Obstetrics and Gynecology, San Paolo Hospital, University of Milan, 20142 Milan, Italy; ^5^Dentistry Chair, Department of Health Sciences, San Paolo Hospital, University of Milan, 20142 Milan, Italy; ^6^Pathology Unit, Department of Medicine, Surgery and Dentistry, Fondazione IRCCS Ca' Granda Ospedale Maggiore Policlinico, 20142 Milan, Italy

## Abstract

The aim of this study was to assess the prevalence of HPV infection and determinants of abnormal cytology in HIV-positive patients. In a cross-sectional study, patients of both sexes, asymptomatic for HPV, underwent anorectal (men)/cervical (women) and oral swabs. Cytology and HPV-PCR detection/genotyping (high- and low-risk genotypes, HR-LR/HPV) were performed. A total of 20% of the 277 enrolled patients showed oral HPV, with no atypical cytology; in men, anal HPV prevalence was 81% with 64% HR genotypes. In women, cervical HPV prevalence was 58% with 37% HR-HPV. The most frequent genotypes were HPV-16 and HPV-18; 37% of men and 20% of women harbored multiple genotypes. Also, 47% of men showed anal squamous intraepithelial lesions (SILs); 6% had high- and 35% low-grade SILs (HSILs/LSILs); 5% had atypical squamous cells of undetermined significance (ASC-US). HR-HPV was independently associated with anal-SIL in men (*P* = 0.039). Moreover, 37% of women showed cervical SIL: 14 ASC-US, 15 LSILs, 4 HSILs, and 1 *in situ* cancer. The presence of both LR and HR-HPV in women was independently associated with SIL (*P* = 0.003 and *P* = 0.0001). HR-HPV and atypical cytology were frequently identified in our cohort. HPV screening should be mandatory in HIV-infected subjects, and vaccine programs for HPV-negative patients should be implemented.

## 1. Introduction

Human papillomavirus (HPV) is one of the most common sexually transmitted pathogens, responsible of several different diseases ranging from benign common warts to invasive carcinoma at a variety of anatomical sites, including the cervix, vulva, vagina, penis, anus, and oropharynx [1].

While the immune system eliminates over time most HPV infections in immunocompetents individuals, HPV infections tend to persist in immunodeficient ones, such as HIV-positive subjects, probably due to the inability to control the expression and replication of HPV by HIV-compromised immune system [2]. Persistent infections with oncogenic HPV genotypes are causally related to the development of high-grade intraepithelial lesions and invasive carcinoma.

Many studies have shown a higher incidence of HPV-related cervical cancer [3] in HIV-positive women compared to general population, and in 1993, cervical cancer was included in the list of AIDS defining conditions [4]. Later, attention was paid on anal cancer and its increasing incidence in HIV-positive patients, especially in men who have sex with men (MSM), when compared with HIV-negative controls [5]. Furthermore, the risk of other HPV-associated cancers, like oropharyngeal, has been demonstrated to be increased among HIV-infected individuals [6].

The effect of highly active antiretroviral therapy (HAART) on the incidence of HPV-associated infection and related neoplasia is controversial. Several studies have indicated that the incidence of anal cancer but not cervical cancer is increased with HAART [7, 8]. At the same time, HAART has not been shown to substantially reduce progression to high-grade cervical intraepithelial neoplasia (CIN) or anal intraepithelial neoplasia (AIN) in HPV-HIV-coinfected individuals [9]. To date, no studies have analyzed HPV infection at oral and genital sites and related dysplasia in the context of effective HAART.

Epidemiological studies on local (Italian) prevalence of HPV cervical infection in HIV-negative women published in recent years showed HPV prevalence ranging from 8.8% [10] to 38% [11] depending on the population examined. In Northern Italy (region in which the present study is done), the prevalence of cervical cancer screening is higher than in other regions, covering 79.5% of women versus 70.9% [12]. Few studies have investigated anal HPV prevalence in HIV-negative population in Italy; a recent study conducted on HIV-negative MSM individuals, attending sexually transmitted infections (STIs) clinic in Rome showed a prevalence of anal HPV infection of around 74% [13]. 

Given these considerations, the aims of our study were to assess the prevalence, genotype diversity, and determinants of HPV infection at different mucosal sites (oral, cervical, and anal) in a cohort of HIV-positive men and women asymptomatic for HPV. Furthermore, we wanted to assess the prevalence of atypical cytology at different sites and to identify any demographic and clinical factors associated with the development of intraepithelial lesions, considered precancerous lesions. 

## 2. Methods 

### 2.1. Study Population

We performed a cross-sectional study in a cohort of HIV-positive men and women followed at the Clinic of Infectious Diseases, Department of Health Sciences, San Paolo Hospital, Milan, between January 2009 and February 2011. Inclusion criteria were HIV positive, age ≥18 years, routinely followed by the clinic, asymptomatic for genital diseases, and signed the informed consent to participate the study. Exclusion criteria were history of anal/cervical cancer, ongoing genital signs and symptoms (discharge, discomfort, or ulceration), and refusal to sign the informed consent. 

Patients were asked to complete an anonymous structured questionnaire on sexual behavior. The questionnaire was not validated; 14 self-reported questions were asked on the number of partners, genital intercourse, oral intercourse, and history of sexually transmitted diseases (STDs) (attached as supplementary document available online at doi: http://dx.doi.org/10.1155/2013/915169).

### 2.2. Surgical, Gynecological, and Dental Visits

During the gynecological and surgical visits, cervical specimens (women) and anal specimens (men) were collected to analyze cytology and detect HPV. Oral specimens were obtained during the dentistry visit for both sexes for the same analyses. 

In particular, the oral sample was obtained by scraping the walls of the oropharyngeal cavity and gums using a dermatologic curette (a scalpel with blade ring and a diameter of 7 mm) with tangential movements at about 30 degrees on the mucosal. Then with the help of a Heidemann spatula, the material collected was smeared on a slide for citologyc analysis. Another sample obtained in the same way was collected in liquid-based cytology medium (PreservCyt-Hologic) for HPV detection and genotyping.

### 2.3. Cytology

Anal, cervical, and oral cytology samples were collected using a Dacron swab to perform a pap smear. The cytology was read by expert cytopathologists, with expertise in oral cytology and classified according to the 2001 Bethesda System terminology as negative, atypical squamous cells of undetermined significance (ASC-US), atypical squamous cells that cannot exclude high-grade SILs (ASC-H), low-grade SILs (LSILs), high-grade SILs (HSILs), or carcinoma [14]. Unsatisfactory oral, cervical, or anal cytology results due to insufficient cellularity were excluded from cytological classification.

### 2.4. HPV Detection and Genotyping

Specimens from the cervical, oral, and anal mucosae were collected in liquid-based cytology medium (PreservCyt-Hologic). Total DNA was extracted using a commercial kit (QIAamp DNA Blood Mini KIT, Qiagen). HPV DNA was detected with PCR using both the L1 consensus primers MY09/MY11 [15] and the E6/E7 consensus primers PU-1M/PU-2R [16]. Viral genotyping was performed using a direct sequencing kit (BD Terminator Kit v 1.1, Applied Biosystems, Life Technologies) on an automated capillary electrophoresis sequencer (ABI3130, Applied Biosystems, Life Technologies). The sequences obtained were blasted against the NCBI nucleotide DNA database using the Basic Local Alignment Search Tool (BLAST: http://blast.ncbi.nlm.nih.gov/) to identify the viral genotype. The oncogenic risk of the different viral genotypes was defined according to the epidemiologic classification by Muñoz et al. [17].

### 2.5. Statistical Analyses

Demographic and immunovirological characteristics of the population were summarized using absolute numbers and percentages for categorical variables and medians and interquartile ranges (IQRs) for continuous variables. 

Categorical and continuous parameters were compared between HPV-negative and HPV-positive patients of the entire study population using Pearson's chi-square test and the Mann-Whitney test.

Two univariate and multivariate logistic regression analyses were performed to explore factors associated with anal (model 1) and cervical (model 2) dysplasia in men and women, respectively. The following variables were included in the model: age, exposure categories for HIV infection (MSM versus no MSM and heterosexual women versus other exposure categories), CDC stage C (according to CDC 1993 revised HIV classification of Center of Diseases and Control of Atlanta; the system is based on 3 ranges of CD4 counts and 3 clinical categories; C category includes all the AIDS defining diseases) [4], ongoing HAART (yes versus no), CD4+ T-cell Nadir count, and presence of HPV (LR and/or HR versusnegative).

The analyses were performed using SPSS software (version 18.0).

## 3. Results

### 3.1. Patient Characteristics

A total of 277 HIV-positive patients were enrolled, and 97 (35.0%) were females. Female prevalence of the study population is consistent with the gender distribution in our clinic, with around 2/3 of patients that were males and 1/3 females, according to Italian data [18]. Demographic variables of the study population were similar to those of all patients attending our clinic (data not shown). The median age was 42 years (interquartile range-IQR: 35–47). Sexual transmission was the most frequent route of infection for both men (136/180 infected by homosexual and 29 by heterosexual intercourse) and women (69/97 infected by heterosexual intercourse), as self-declared by the patients during the first visit at the clinic and reported in the medical history charts. Only 47 patients (17%) were in CDC group C; 222 (80.1%) were on HAART. Women had a longer duration of documented HIV infection (median, IQR: 143, 55–210 versus 52, 24–411 months; *P* = 0.0001), lower CD4 counts at Nadir (median, IQR: 205 cells/mmc, 120–288 versus 273 cells/mmc, 163–397; *P* = 0.003), and a longer duration of HAART (median, IQR: 70, 33–152 versus 44, 22–130.5 months; *P* = 0.05) compared with men. Demographic characteristic of the study population according to HPV infection status are shown in Table 1. A total of 117 (65.0%) men and 36 (37.1%) women completed the questionnaire on sexual behavior, and 60% reported a positive history of STDs. Men declared more than 25 sexual partners lifelong (61/117 versus 10/36; *P* = 0.01) and anal intercourse (98/117 versus 14/36; *P* < 0.0001) more frequently than women; oral intercourse was reported by 87% of patients (see Table 2). A total of 44% of the 277 enrolled patients did not complete the survey.

### 3.2. Prevalence of HPV at Anal, Cervical, and Oral Sites

Each subject underwent a surgical (men) or gynecological (women) visit; 201 patients (72%) underwent also a dental visit (61 women and 140 men). The subjects were analyzed for both HPV infection and abnormal cytology at genital and oral sites. 

A total of 40 (20%) tested individuals harbored HPV infection at the oral site, and 18 (9.0%) had HR-HPV genotypes. The overall prevalence of anal HPV in men was 81% (145/180 men), and HR-HPV was detected in 114/145 of them (78.6%). Cervical HPV was detected in 56 (58%) of the 97 tested women, and 36/56 (64.3%) harbored HR-HPV. Globally, HPV was detected at genital sites (cervical in women and anal in men) in 201 individuals (72.6% of the cohort), and 150/201 (74.6%) harbored HR types.

A total of 78/201 subjects (38.8%) tested at both genital and oral sites showed concordant HPV serostatus (HPV positive in 32 cases and HPV negative in 46 cases); positivity at only the oral site was found in 7 cases (3.5%), and HPV was only detected at genital sites in 116 cases (57.7%). 

Overall, the subjects harboring HPV at any site were more frequently men (men represented 72% of HPV-positive and 35% of HPV-negative subjects; *P* = 0.0001) and more frequently infected through sexual routes (homo- and heterosexual intercourses accounted for 89% of HPV-positive cases versus 68% of HPV-negative cases; *P* = 0.0001). HPV-coinfected individuals were at a less advanced stage of HIV disease than HPV-negative ones, as documented by the shorter duration of HIV infection (median, IQR: 68 months, 29–169 versus 120 months, 39–181; *P* = 0.028) and higher nadir CD4 cell counts (median, IQR: 271 cells/mmc, 157–392 in HPV positive versus 209 cells/mmc, 139–287; *P* = 0.028). Current HIV RNA copy levels were higher in HPV-positive subjects (median, IQR HIV RNA log10 copies/mL: 1.77, 1.59–3.25 versus 1.59, 1.59–1.79; *P* = 0.0002). Atypical cytology was diagnosed in 18/76 (23.7%) HPV-negative and 99/201 (49.2%) HPV-positive subjects (*P* = 0.0001) (Table 1).

 Among males completing the questionnaire, having more than 25 sexual partners, anal and oral intercourses were more frequent in HPV-positive individuals than HPV-negative ones. Among women, those with HPV cervical infection had a higher occurrence of other STDs (Table 2). 

### 3.3. Representation of HPV Genotypes at Anal, Cervical, and Oral Sites

**Figure 1:**
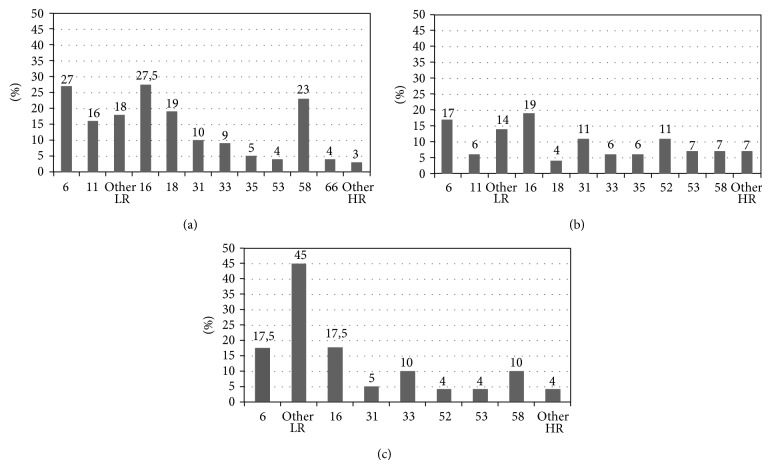
Prevalence of different HPV genotypes at the anal site (a), cervical site (b), and oral site (c). (a) The most frequent HPV genotypes are listed on the abscissa. Genotypes 6 and 11 are low risk, and other LR indicates other low-risk genotypes. Genotypes 16, 18, 31, 33, 35, 53, 58, and 66 are high risk, and other HR indicates other high-risk genotypes. The percentage indicates the prevalence of each genotype within the total HPV genotypes in the HPV-positive population. (b) The most frequent HPV genotypes are listed on the abscissa. Genotypes 6 and 11 are low risk, and other LR indicates other low-risk genotypes. Genotypes 16, 18, 31, 33, 35, 52, 53, and 58 are high risk, and other HR indicates other high-risk genotypes. The percentage indicates the prevalence of each genotype within the total HPV genotypes in the HPV-positive population. (c) The most frequent HPV genotypes are listed on the abscissa. Genotypes 6 and 11 are low risk, and other LR indicates other low-risk genotypes. Genotypes 16, 31, 33, 52, 53, and 58 are high risk, and other HR indicates other high-risk genotypes. The percentage indicates the prevalence of each genotype within the total HPV genotypes in the HPV-positive population.

The prevalence of different HPV genotypes at anal, cervical, and oral sites is shown in Figure 1. HR genotypes 16 and 18 were detected in 27% and 19% of males and 18% and 3% of females, respectively. The other frequently detected genotypes were genotype 6 (accounting for 26% of HPV-positive men and 16% of women), genotype 58 (in 23% of men), and genotype 52 (in 10% of women). HPV 16 was detected in 36% of those individuals found to be HPV positive at the oral site. 

Overall, men were more likely to harbor genotypes 11 (16% versus 5.4%;  *P* = 0.049), 18 (19.45 versus 3.6%; *P* = 0.005), and 58 (23.6% versus 7.2%; *P* = 0.009), whereas there were no differences in the distribution of other LR and HR genotypes according to gender.

Up to 37% of men and 20% of women showed multiple genotypes. Patients with HR-HPV more often harbored multiple HPV strains compared with those with LR-HPV (78/144 multiple in HR versus 7/48 in LR; *P* = 0.0001).

### 3.4. Prevalence and Predictors of Atypical Cytology

Data on the occurrence of atypical cytology, which were defined as ASC-US, ASC-H, LSILs, and HSILs, and related variables were examined in anal (men) and cervical (women) specimens. A total of 7/180 anal specimens (3.8%) and 3/97 (3%) cervical specimens were unsatisfactory for cytologic classification and were excluded from the analyses. 

No abnormal cytology was found at the oral site. At the anal site, a total of 83 patients (47%) showed atypical cytology; 11 patients (6% of total) had HSILs, 62 (35%) had LSILs, and 10 (5%) had ASC-US. A total of 76/83 men with atypical cytology (91.5%) were HPV positive, and 63 (76%) harbored HR-HPV strains. HR-HPV was more frequently detected in men with more severe dysplasia (HR-HPV: 91% among 11 subjects with HSILs, 79% among 62 with LSILs, 60% among 10 with ASC-US, and 53% among 92 with normal cytology; *P* = 0.0002). Two variables were associated with a higher risk of atypical cytology in a univariate analysis: being infected through the homosexual route (odds ratio (OR): 4.05; 95%CI: 1.88–8.69) and harboring HR-HPV (OR: 5.23; 95%CI: 2.08–13.17 versus HPV negative). Advanced age (OR: 0.95, 95%CI: 0.92–0.98 for each year older) and being on HAART (OR: 0.44, 95%CI: 0.22–0.88 versus HAART naïve) were associated with a lower risk. After controlling for demographic and HIV-related factors (age, exposure categories for HIV infection, CDC stage C, HAART, CD4+ T-cell Nadir count, and presence of HPV infection), only subjects harboring HR-HPV remained significantly at risk for atypical cytology compared with HPV-negative individuals (adjusted odds ratio (AOR): 3.48; 95%CI: 3.54–20.47) (Table 3(a)). 

Regarding cervical findings, 34 (37%) women showed abnormal cervical cytology (13 had ASC-US, 1 had ASC-H, 15 had LSILs, 4 had HSILs, and 1 had a diagnosis of *in situ* squamous cell cancer). Of these 34 women, 29 (85%) were HPV positive, and 20 (59%) harbored HR-HPV genotypes. HR-HPV was detected more frequently in women with more severe atypical cytology (HR-HPV: 100% in women with HSILs, 60% in patients with LSILs, 69% in patients with ASCUS, and 24% women with normal cytology; *P* = 0.0001). In the univariate model, women infected via the sexual route were more likely to have atypical cytology (OR: 4.05, 95% CI: 1.88–8.70 versus no heterosexual infection); no other demographic or clinical characteristics were associated with occurrence of atypical cytology. The detection of both LR-HPV and HR-HPV was associated with atypical cytology (OR: 10.80, 95%CI: 2.45–47.57 and OR: 18.86, 95%CI: 4.86–73.11, resp., versus HPV negative). In the multivariate model, only the presence of either LR-HPV or HR-HPV was confirmed to be associated with atypical cytology (OR: 18.00, 95%CI: 2.75–117.9 and OR: 26.86, 95%CI: 5.19–138.62, resp., versus HPV negative) (Table 3(b)).

Overall, men showed a nonstatistically higher risk of SILs compared with women (83 out of 180 versus 34 out of 97, resp., *P* = 0.09).

## 4. Discussion

In this study, we analyzed the contemporary prevalence of HPV in both sexes at different sites (genital and oral) in a population of HIV-positive individuals in a high-income country. 

The overall prevalence of HPV infection in our cohort was 72.6% (including the anal site in men and cervical site in women); HPV was more frequent in men than in women (81% versus 58%), and oral localization was detected in up to 20% of individuals. 

Our results are consistent with those of previous studies conducted in HIV-positive populations, in which the prevalence rates of HPV infection were 48%–76.9% [19, 20] in women and 60%–89% [21, 22] in men. Moreover, among men, as widely described in the literature [13, 23], HPV infection at anal site is more frequent in men who have sex with men compared to those with other risk factors for HIV. Data from the literature show that HIV-positive MSM displays a 60-fold higher risk than heterosexual men for the development of anal cancer [24].

As expected, these prevalence rates are higher than those observed in the general population [25–27]. Many factors have been proposed to explain the high HPV prevalence in HIV-positive subjects. HIV infection could lead to an increased risk of reactivation of latent infections, and the persistence of HPV infection could be due to immune system dysregulation [28]. In addition, the higher HPV prevalence in HIV-positive population could reflect more active screening procedures for HPV in comparison to HIV-negative subjects; the policy of active screening for different diseases in HIV positive population is consistent with the evidence of being at risk of several infectious conditions because of immunedepression. Moreover, recent studies have shown that infection with oncogenic HPV genotypes could multiply the risk of acquiring HIV [29]. In fact, in one recent study, cervicovaginal HPV infection with HR genotypes was associated with an increased risk of HIV acquisition in women [30].

The estimates of oral HPV prevalence in the literature are highly variable [31], and it is difficult to discern whether such variability reflects methodological or population differences. The 20% prevalence of oral HPV infection identified in our study is similar to previous data on HIV-infected subjects [6, 21]. The finding of 7 cases (3.5% of the total) with HPV infection only at the oral site might reflect oral sexual behavior, which should be taken into account in epidemiological and clinical studies, even if transmission route of HPV infection is still unclear and other route than sexual are possible.

 In terms of viral genotypes, it is interesting to note that no concordance in HPV genotype was found in our cohort in concomitant infections at both oral and genital sites, which occurred in 38.8% of HPV-positive individuals, and this result is consistent with previous studies [20, 21]. Different susceptibilities to HPV infection of the oral and genital mucosae might explain the differences in HPV genotype distribution [32]. 

Regarding the immunovirological profile of our cohort according to HPV serostatus, we observed that HPV-coinfected individuals showed higher median CD4 T-cell counts compared with HPV-negative subjects. These findings are in contrast with other studies [21] and might reflect a selection bias because the majority of subjects that we screened were “well-being” patients, that is, with better immunological status and without severe comorbidities. The association between higher viral load and HPV infection is in line with a previous study by Strickler et al., which confirmed that high HIV viral load was strongly associated with the detection of HPV in women showing either a high (>500 cells per mm^3^) or moderate (200–500 cells per mm^3^) CD4 T-cell count but not in women with a low CD4 T-cell count [33]. It could be suggested that nonadherent individuals with a high HIV viral load have more risky sexual habits, leading to an increased likelihood of acquiring HPV. 

We did not find any associations between HAART usage and HPV infection. Data regarding the impact of HAART on HPV infection and HPV-related cervical abnormalities are inconsistent or controversial [9, 34]. The lack of association between HAART and HPV in our cohort is consistent with evidence that the incidence of HPV-related malignancies in HIV-infected subjects has not declined with the introduction of potent antiretroviral therapy [9, 35]. 

High-risk HPV genotypes are associated with the development of cancer [36]. Globally, the HR-HPV anal prevalence in our cohort is lower than the data reported by Conley et al. [22] (possibly because we selected asymptomatic patients) and similar to that reported in a recent study by Parisi et al. [21] conducted on HIV-infected patients from the same geographic area (i.e., Northern Italy). 

Regarding cytological data, only 3% of the total specimen was not appropriate for diagnoses; this percentage is very low and could not affect the results, in our opinion. Concerning anal dysplasia, SILs were diagnosed in 47% of men; our results are in line with those of previous reports showing a 34%–71% prevalence of abnormal anal cytology [31, 37, 38]. The univariate predictors of SILs among men were MSM, younger age, and infection with HR-HPV; HAART showed a protective role in SIL development. In the multivariate model, only HR-HPV infection was confirmed to be significantly associated with dysplasia. Interestingly, a recent study confirmed the lower prevalence of HPV infection and anal SILs in heterosexual men compared with MSM [39]. The observed association between younger age and dysplasia in male subjects is not confirmed in other reports on HIV-negative males [40]. Together with other authors [25, 40], nevertheless, we speculate that a larger number of sexual encounters (in younger as well as MSM individuals) might result in infection with greater number of HPV types, and this could potentially increase exposure to other unmeasured risk factors, associated with anal intercourse, and might also reflect a greater number of mucosal lesions. 

Both LR-HPV and HR-HPV genotypes were associated with atypical cytology in women of our cohort, with an 85% prevalence of HPV infection in 59% of cases characterized by HR genotypes. Data available on Italian women routinely screened for cervical dysplasia suggest that the prevalence of HR-HPV and LR-HPV infection is lower than in our cohort [41]. 

Notably, up to 53% of men and 24% of women harbor HR-HPV without any cytologic damage; further studies are required to better evaluate this population of healthy HR-HPV carriers. The absence of oral mucosal abnormalities despite a 20% prevalence of HPV infection at the oral site is in contrast with recent studies in which HIV-positive individuals more frequently displayed an oral mucosal abnormality [6] than the general population. In fact, HPV is etiologically associated with a subset of head and neck squamous cell carcinomas (HNSCCs) [42, 43]. The absence of oral dysplasia in our cohort might be explained by different methods of collecting mucosal material (cytobrushing instead of biopsy) or by the site of collection (oral cavity versus tonsillar epithelium). Even if it must be said that one of the limits of the present study is represented by the modest sample size, which might affect the generalizability of our results, we can draw several conclusions. 

The main findings from this cohort study are the high prevalence of HPV and high-risk HPV genotypes in our cohort of HIV-positive men and women who are asymptomatic for genital distress. Of major concern is that almost half of the subjects suffer from genital or anal dysplasia with different degrees of severity. 

The long-term followup of subjects with dysplastic lesions will clarify their possible evolution in the HIV setting. On the basis of these results, we suggest mandatory screening for both HPV and related lesions in all HIV-positive subjects and the possible implementation of vaccination studies and programs in HIV-positive/HPV-negative patients.

## Supplementary Material

Questionnaire on sexual behavior: self-reported questions on number of sexual partners, type of sexual intercourse and history of sexually transmitted diseases.

## Figures and Tables

**Table 1:** Demographic characteristics of the study population according to HPV infection status.

Characteristics	HPV negative (76)	HPV positive (201)	*P*
Age∗	42.5 (38–49)	41 (35–47)	0.119
Male°	35 (46%)	145 (72%)	**0.0001**
Exposure categories°			**0.0001**
MSM	17 (22%)	117 (58%)	
Heterosexual	35 (46%)	62 (31%)	
IDUs	23 (31%)	22 (10%)	
Vertical	1 (1%)	0	
CDC C°	18 (24%)	29 (14%)	0.067
HIV duration (mths)∗	120 (39–181)	68 (29–169)	**0.028**
CD4+ T cells/mmc∗	462 (346–636)	480 (357–665)	0.710
Nadir CD4+ T cells/mmc∗	209 (139–287)	271 (157–392)	**0.006**
Log_10_ HIV RNA cp/mL∗	1.59 (1.59–1.79)	1.77 (1.59–3.25)	**0.002**
HAART°	63 (83%)	159 (79%)	0.480
HAART duration (mths)∗	60 (40–133)	44 (22–123)	0.081
Dysplasia°	18 (23.7%)	99 (49.2%)	**0.0001**

°Data are presented as the number (percentage). ∗Data are presented as the median (IQR).

Exposure categories for HIV infection: MSM: men who have sex with men; IDUs: injection drug users; CDC C: AIDS classification according to Atlanta CDC 1993; HAART: highly active antiretroviral therapy.

**Table 2:** Data from sexual behavior questionnaires according to gender and to HPV infection status.

Questionnaire	HPV negative	HPV positive	*P*
Males (*n* = 117)	(*n* = 28)	(*n* = 89)	

STDs° (*n* = 73, 62%)	*n* = 15 (53%)	*n* = 58 (65%)	0.26
>25 partners° (*n* = 61, 52%)	*n* = 7 (25%)	*n* = 54 (61%)	**0**.**001**
Anal intercourse° (*n* = 98, 83%)	*n* = 17 (60%)	*n* = 81 (91%)	**0**.**0001**
Oral intercourse° (*n* = 105, 89%)	*n* = 21 (75%)	*n* = 84 (94%)	**0**.**0032**

Females (*n* = 36)	(*n* = 10)	(*n* = 26)	

STDs° (*n* = 19, 52%)	*n* = 2 (20%)	*n* = 17 (65%)	**0**.**014**
>25 partners° (*n* = 10, 27%)	*n* = 2 (20%)	*n* = 8 (31%)	0.51
Anal intercourse° (*n* = 14, 38%)	*n* = 3 (30%)	*n* = 11 (42%)	0.49
Oral intercourse° (*n* = 28, 77%)	*n* = 8 (80%)	*n* = 20 (77%)	0.84

STDs: sexually transmitted diseases.

**Table:** (a) Factors associated with anal atypical cytology

	Univariate			Multivariate		
	OR	95%CI	*P*	AOR	95%CI	*P*
Age (each year more)	0.953	0.923–0.984	0.003	0.964	0.928–1.002	0.065
Exposure categories MSM (versus no MSM)	4.046	1.883–8.696	0.0001	2.200	0.892–5.426	0.087
CDC C (yes versus no)	0.674	0.296–1.537	0.348	0.489	0.135–1.775	0.277
HAART (versus naive)	0.440	0.219–0.881	0.020	0.549	0.195–1.542	0.255
Nadir CD4+ T cells (each unit more)	1.000	0.998–1.002	0.945	0.999	0.996–1.001	0.355
HPV infection						
HPV negative		Ref			Ref	
LR versus HPV negative	2.731	0.973–8.257	0.075	2.830	2.222–17.036	0.139
HR versus HPV negative	5.233	2.078–13.175	0.0001	3.479	3.543–20.467	0.039

MSM: men who have sex with men; CDC C: AIDS classification according to Atlanta CDC 1993; HAART: highly active antiretroviral therapy; LR: low-risk genotypes; HR: high-risk genotypes.

**Table:** (b) Factors associated with cervical atypical cytology

	Univariate			Multivariate		
	OR	95%CI	*P*	AOR	95%CI	*P*
Age (each year more)	1.000	0.954–1.048	0.992	0.975	0.907–1.047	0.484
Exposure categories Heterosexual (versus others)	4.046	1.883–8.696	**0.0001**	3.332	0.782–14.200	0.104
CDC C (yes versus no)	1.315	0.470–3.679	0.502	2.219	0.445–11.066	0.331
HAART (versus naive)	0.556	0.130–2.383	0.429	0.298	0.030–2.941	0.300
Nadir CD4+ T cells (each unit more)	1.002	0.998–1.005	0.367	1.000	0.996–1.004	0.990
HPV infection						
HPV negative		Ref			Ref	
LR versus HPV negative	10.800	2.452–47.568	**0.002**	17.999	2.748–117.9	**0.003**
HR versus HPV negative	18.857	4.864–73.109	**0.0001**	26.863	5.195–138.622	**0.0001**

CDC C: AIDS classification according to Atlanta CDC 1993; HAART: highly active antiretroviral therapy; LR: low-risk genotypes; HR: high-risk genotypes.
