# Supplementary material for: HPV Infection in a Cohort of HIV-Positive Men and Women: Prevalence of Oncogenic Genotypes and Predictors of Mucosal Damage at Genital and Oral Sites
Source: J Sex Transm Dis. 2013 Mar 5;2013:915169. doi: 10.1155/2013/915169 (PMC4437419; doi:10.1155/2013/915169)
Supplement: Supplementary file 1 — Questionnaire on sexual behavior: self-reported questions on number of sexual partners, type of sexual intercourse and history of sexually transmitted diseases. [file 915169.f1.doc]

Attached 2

**QUESTIONNAIRE ON SEXUAL BEHAVIORS**

**This questionnaire will be identified by a non-nominal code**

### **Patient Code** |__|__|__|__|__|__|__|

### **Compilation Date**|__|__||__|__| |__|__|

day month year

***Sex*** *🞎* ***F*** *🞎* ***M***

***Date of birth***|__|__|__|__| ***Nation of birth*** _________________________

***Have you ever had sexual intercourse?***

*🞎 YES 🞎 NO* ***If your answer is NO, please go to question ***
*How old were you when you had your first sexual intercourse?*** |__|__|

***How would you define your sexual preferences?***

*🞎 Heterosexual 🞎 Homosexual 🞎 Bisexual*

***Have you ever had sexual intercourse with a same-sex person?***

🞎 YES 🞎 NO

**If yes:**

🞎 Once 🞎 Rarely 🞎 Regularly

***How many sexual partners have you had in your life?***

*🞎 None 🞎 One 🞎 From 2 to 3
🞎 From 4 to 5 🞎 From 6 to 10 🞎 From 11 to 25
🞎 More than 25*

***How many sexual partners have you had in the last 12 months?****🞎 None 🞎 One 🞎 From 2 to 3
🞎 From 4 to 5 🞎 From 6 to 10 🞎 From 11 to 25
🞎 More than 25*

***Do you have a stable partner (considered as your main partner, with whom you’ve had a relationship for at least 3 months)?***

*🞎 YES 🞎 NO*

***Do you have occasional sexual intercourse?***

*🞎 YES 🞎 NO*

***Do you use condoms during a sexual intercourse?***

*🞎 Never 🞎 Sometimes 🞎 Always 🞎 I don’t remember*

***Have you ever had anal sexual intercourse?****🞎 YES 🞎 NO*

***If yes:***

*🞎 Once 🞎 Rarely 🞎 Regularly*

***Have you ever had oral sex?****🞎 YES 🞎 NO*

***If yes:***

*🞎 Once 🞎 Rarely 🞎 Regularly*

*****Do you take, or have you ever taken hormone preparations?****🞎 YES 🞎 NO*

***If yes, how long have you been taking them (years)?***

*🞎 <5 🞎 >5 but <10 🞎 >10*

**Have you ever been diagnosed with one of the following genital infections?
*Syphilis*** *🞎 YES 🞎 NO* ***Gonorrhoea*** *🞎 YES 🞎 NO* ***Chlamydia*** *🞎 YES 🞎 NO* ***Mycoplasma*** *🞎 YES 🞎 NO* ***Wart*** *🞎 YES 🞎 NO* ***Condyloma*** *🞎 YES 🞎 NO*

***Other infections?* _____________________________________________**

### **THANK YOU FOR YOUR COOPERATION**
